# Balancing Health, Sustainability, and Food Assurance in Fast-Food Choice: A Discrete Choice Experiment in Taiwan

**DOI:** 10.3390/foods15203679

**Published:** 2026-10-16

**Authors:** Chun-Chieh Ma, Hsiao-Ping Chang

**Affiliations:** 1 Department of Public Administration and Management, National University of Tainan, No.33, Sec. 2, Shu-Lin St., Tainan 70005, Taiwan; ccma@mail.nutn.edu.tw; 2 Department of Health Industry Technology Management, Chung Shan Medical University, No.110, Sec. 1, Jianguo N. Rd., Taichung City 40201, Taiwan; 3 Department of Medical Management, Chung Shan Medical University Hospital, No.110, Sec. 1, Jianguo N. Rd., Taichung City 40201, Taiwan

**Keywords:** discrete choice experiment, consumer food choice, fast food, willingness to pay, nutrition labeling, food traceability, sustainable food consumption

## Abstract

Fast-food choices increasingly involve trade-offs among health, sustainability, food assurance, and price. This study employed a discrete choice experiment to examine consumer preferences and willingness to pay for health-related, sustainability-related, food-assurance, and monetary attributes among 401 respondents in Taiwan recruited through an online convenience-sampling approach in August 2025. The experiment included nutrition labeling, a health-oriented cue, carbon labeling, food traceability, an alternative-food option, and a price premium. Choices were analyzed using conditional logit and random parameter logit models, and marginal willingness to pay (MWTP) was estimated for each non-price attribute. All five non-price attributes significantly increased choice utility, whereas the price premium had a significant negative effect. The highest MWTP point estimate was observed for nutrition labeling (TWD 12.63), followed by the health-oriented cue (TWD 11.76), food traceability (TWD 10.59), carbon labeling (TWD 8.05), and the alternative-food option (TWD 7.84). This ordering should be interpreted descriptively because pairwise statistical differences among the MWTP estimates were not formally tested. Random parameter logit estimates were broadly consistent with the conditional logit results, with significant unobserved heterogeneity detected only for the conventional alternative-specific constant. Overall, the findings indicate that respondents assigned positive value to health-, sustainability-, and food-assurance attributes while remaining sensitive to additional cost, supporting the joint consideration of these attributes when examining consumer preferences in fast-food choice.

## 1. Introduction

Food choice is increasingly shaped by multiple product attributes that extend beyond taste, convenience, and price. Nutritional quality, food safety, traceability, and environmental sustainability have become increasingly salient characteristics of the foods consumers evaluate and purchase. This convergence of human and planetary health has stimulated growing interest in sustainable healthy diets, in which food choices are assessed not only in terms of individual health benefits but also their broader environmental implications. A recent systematic review of 57 studies showed that health and sustainability have become increasingly interconnected in consumer food-choice research, particularly in relation to meat reduction, plant-based foods, and alternative proteins [1]. Nevertheless, consumers may not assign equal importance to health, sustainability, and other food-related attributes, and understanding the trade-offs among these characteristics remains important for explaining food choice.

This issue is particularly relevant in fast-food settings, where consumers traditionally make choices based on convenience, affordability, and rapid service but are increasingly exposed to nutrition and environmental information. Previous research suggests that nutrition labeling can influence consumer awareness and food selection, while climate-impact and environmental labels can also affect restaurant and fast-food choices [2,3,4,5]. These findings indicate that both health-related and sustainability-related information may contribute to consumer evaluations in fast-food contexts.

Consumer valuation of sustainability- and food-assurance-related attributes is also reflected in willingness-to-pay research. Evidence from discrete choice studies indicates positive, although heterogeneous, willingness to pay for environmental sustainability labels and food traceability [6,7]. Research on plant-based and other meat-alternative products further shows that nutritional, environmental, and product information can influence consumer preferences and willingness to pay [8,9,10]. These findings provide relevant context for examining a broader alternative-food attribute alongside nutrition, sustainability, traceability, and price within a common fast-food choice framework.

Despite this growing literature, much of the existing evidence has examined individual food-labeling interventions, specific sustainability claims, food-traceability attributes, or meat-alternative products separately. Consequently, comparatively less attention has been devoted to how consumers simultaneously trade off multiple food attributes representing health, environmental sustainability, food assurance, and monetary cost within a single fast-food choice context. This distinction is important because food choices are typically made among bundles of attributes rather than through isolated evaluations of individual product characteristics. Discrete choice experiments (DCEs) are particularly suitable for examining such trade-offs because they require respondents to choose among alternatives composed of multiple attributes, thereby revealing the relative utility attached to each attribute and allowing marginal willingness to pay to be estimated. A recent systematic review published in *Foods* identifies DCEs as an important econometric approach for measuring sustainable food preferences and willingness to pay while emphasizing the need to account for preference heterogeneity [11].

Against this background, the present study investigates how Taiwanese consumers balance and value multiple food attributes when choosing among fast-food options. A discrete choice experiment was designed around six attributes: nutrition labeling, a health-oriented cue, carbon labeling, food traceability, an alternative-food option, and a price premium. Consumer preferences were estimated using conditional logit and random parameter logit models, allowing both average preferences and unobserved preference heterogeneity to be examined. The study has three objectives: (1) to estimate consumers’ preferences for health-, sustainability-, and food-assurance attributes in fast-food choice; (2) to quantify the marginal willingness to pay for each attribute and thereby examine their relative valuation under a common monetary trade-off; and (3) to investigate heterogeneity in consumer preferences for selected food attributes. By evaluating nutrition, health, environmental sustainability, traceability, alternative-food, and price attributes within a single fast-food choice framework, this study provides a direct comparison of how consumers value multiple food-related benefits under common choice conditions. This integrated approach extends previous research that has typically examined these food attributes separately and provides evidence on how multiple food-related attributes are jointly valued within a fast-food choice context.

## 2. Literature Review and Hypothesis Development

### 2.1. Multiple Food Attributes in Fast-Food Choice

Food choice is a multidimensional decision in which consumers evaluate combinations of sensory, nutritional, economic, safety-related, and environmental attributes. Contemporary research has increasingly emphasized the interconnected roles of human health and planetary health in dietary decision-making. In a systematic review of sustainable healthy food consumption, Bourke et al. [1] concluded that health and sustainability considerations increasingly coexist in consumer decision-making, although translating these concerns into actual food choices remains challenging.

Importantly, health and environmental sustainability do not necessarily receive equal weight in consumers’ decisions. Recent evidence demonstrates substantial heterogeneity in the way individuals make trade-offs among healthiness, sustainability, naturalness, and price. Using a discrete choice experiment, Fantechi et al. [12] identified distinct consumer segments: some simultaneously valued health and sustainability, others prioritized health, whereas another substantial segment based decisions primarily on price. Similarly, experimental research examining the simultaneous presentation of nutrition and environmental labels has shown that health and sustainability information may interact in consumers’ perceptions rather than being processed independently. Jürkenbeck et al. [13], for example, found that Nutri-Score and Eco-Score information influenced consumers’ perceptions of both product healthiness and environmental friendliness.

These trade-offs are particularly relevant to fast-food consumption. Fast food has traditionally competed on convenience, taste, accessibility, and price; however, the sector is increasingly exposed to demands for healthier products and lower environmental impacts. Recent experimental evidence indicates that sustainability information can meaningfully affect choices even within fast-food environments. Wolfson et al. [4] demonstrated that climate-impact menu labels influenced fast-food ordering choices, while a subsequent randomized clinical trial by Wolfson et al. [5] showed that traffic-light-style ecolabels reduced the greenhouse-gas emissions associated with full fast-food meal orders and, in some restaurant contexts, also improved nutritional quality.

The coexistence of health and sustainability concerns therefore raises an important question: when consumers are simultaneously presented with nutrition, health, environmental, food-assurance, and monetary attributes, how do they trade these benefits against one another? This issue is particularly suited to a discrete choice framework because consumers must choose among bundles of competing attributes rather than evaluate each characteristic in isolation. Recent methodological reviews confirm that DCEs remain an important approach for identifying trade-offs and estimating willingness to pay in sustainable food research [11].

Accordingly, the present study conceptualizes fast-food choice as a trade-off among three non-monetary food-related dimensions—health-related attributes, sustainability-related attributes, and food assurance—together with a monetary price premium.

### 2.2. Health-Related Attributes in Fast-Food Choice

#### 2.2.1. Nutrition Labeling

Nutrition information reduces consumers’ uncertainty regarding the nutritional characteristics of food and may assist individuals in identifying options that better correspond to their dietary goals. Nutrition labeling is therefore particularly relevant in restaurant and fast-food settings, where consumers typically make decisions within a relatively short period and nutritional composition may not otherwise be readily observable.

A recent systematic review of restaurant menu nutrition information found that providing nutrition information can influence consumer awareness, purchase intentions, nutrient selection, and calorie-related decisions, although effects vary considerably across studies and consumer segments [2]. Abbas and Hatch [2] specifically concluded that the effectiveness of restaurant nutrition information depends on contextual and sociodemographic conditions and is not uniform across consumers. At the product level, Kelly et al. [3] similarly concluded that nutrition labeling can support healthier purchasing decisions, particularly when information is presented in accessible and interpretable formats.

This evidence is consistent with the original survey design, in which nutrition labeling informed respondents about energy, protein, fat, saturated fat, trans fat, sugar, carbohydrate, and sodium. Because such information makes otherwise difficult-to-observe nutritional characteristics more salient at the point of choice, the presence of nutrition labeling is expected to increase the utility associated with a fast-food alternative.

**H1.**  *The presence of nutrition labeling positively influences consumers’ utility for a fast-food option.*

#### 2.2.2. Health-Oriented Cue

Health-related cues can influence how consumers evaluate food products by making perceived health benefits more salient at the point of choice. Previous research shows that consumers are often willing to pay a premium for healthier foods [14], while food-related knowledge and attitudes are associated with willingness to pay more for healthier options [15]. In restaurant settings, health-related information and health-oriented positioning may also shape consumers’ use of menu information and their evaluations of healthier food offerings [16,17].

An important operational distinction is required in the present study. In the original questionnaire, “health orientation” was described in terms of personal health attitudes, beliefs, and behaviors; however, within the discrete choice experiment, this information was presented as a binary product-level attribute that could be present or absent in Fast-food Options A and B. It therefore does not represent a validated psychometric measure of individual health consciousness. To avoid such an interpretation, the present manuscript refers to this attribute as a health-oriented cue. Accordingly, the HO coefficient is interpreted as the utility associated with the presence of a general health-related product cue rather than as the effect of an individual-level health-orientation construct or a standardized health claim.

Given prior evidence that consumers may attach positive value to health-related food information and positioning, the following hypothesis is proposed:

**H2.**  *The presence of a health-oriented cue positively influences consumers’ utility for a fast-food option.*

### 2.3. Sustainability-Related Attributes in Fast-Food Choice

#### 2.3.1. Carbon Labeling

Environmental impacts associated with food production are largely invisible to consumers at the point of purchase. Carbon and climate labels attempt to reduce this information gap by communicating the greenhouse-gas implications of food choices. In the original questionnaire, the carbon-label attribute was described as information concerning a product’s contribution to global warming and its carbon footprint, with the intention of facilitating more environmentally friendly choices.

Recent empirical evidence provides increasingly strong support for the behavioral relevance of such information. Wolfson et al. [4] found that climate-impact labels altered hypothetical fast-food orders among U.S. adults. In a larger 2026 randomized clinical trial involving 6210 adults, Wolfson et al. [5] showed that traffic-light-style ecolabels resulted in meal choices with substantially lower greenhouse-gas emissions than the control condition across two fast-food restaurant formats.

Evidence on economic valuation also points in the same direction. Bastounis et al. [6] synthesized 43 discrete choice experiments involving 41,777 participants and found that consumers were, on average, willing to pay a premium for foods carrying environmental sustainability labels, although the magnitude varied substantially across products, labels, and populations. Taken together, these findings suggest that carbon-label information may increase the utility of food options by making otherwise difficult-to-observe environmental consequences more salient to consumers.

**H3.**  *The presence of carbon labeling positively influences consumers’ utility for a fast-food option.*

#### 2.3.2. Alternative-Food Options

Alternative foods developed through innovations in food sources, production processes, or manufacturing technologies have received increasing attention as potential contributors to healthier and more sustainable food systems. Meat alternatives may simultaneously convey environmental and health-related benefits, but consumer acceptance remains heterogeneous. Consequently, preferences for alternative foods depend not only on their objective environmental characteristics but also on consumers’ familiarity, perceptions, information exposure, and attitudes toward novel foods.

Recent evidence supports the importance of information in determining the economic valuation of meat alternatives. Chen et al. [8] found that nutritional and environmental information significantly influenced consumers’ willingness to pay for plant-based and cultured meat. Similarly, Jiang et al. [9], using a discrete choice experiment and random parameter logit model, reported that providing environmental information increased preferences and WTP for selected prepared plant-based meat products, while also revealing substantial preference heterogeneity.

Research directly related to fast food suggests that consumers often perceive healthier and more sustainable burger-component alternatives as healthier, but taste judgments may weigh more heavily in acceptance decisions [18]. Consumer responses may also depend strongly on how alternatives are framed. A large U.S. experiment found that meat- and dairy-free foods were more likely to be selected when framed as “healthy” or “sustainable” than when labeled simply as “vegan” or “plant-based,” illustrating the importance of consumer interpretation of alternative-food cues [19].

The original survey defined the substitute-food attribute broadly as innovations in food sources, production processes, and manufacturing technologies intended to improve health, promote sustainable resource use, and reduce environmental pollution, with examples including artificial meat, vegetarian burgers, and meat substitutes. Accordingly, the present manuscript uses the broader term alternative-food option rather than restricting the original attribute retrospectively to plant-based meat alone.

**H4.**  *The presence of an alternative-food option positively influences consumers’ utility for a fast-food option.*

### 2.4. Food Traceability and Food Assurance

Food traceability differs conceptually from direct health and environmental claims. Its primary function is to make information regarding product origin, production, safety, quality, and supply-chain processes more transparent. The original questionnaire similarly described traceability-certified ingredients as providing assurance regarding origin, safety, and quality.

The growing emphasis on food safety, authenticity, and supply-chain transparency has increased consumers’ valuation of traceability systems. Tran et al. [7], in a meta-analysis of 72 peer-reviewed studies, reported an overall upward trend in WTP for traceable food and estimated an average price premium of approximately 32%, although valuation varied across food categories, countries, and information formats. Importantly, the study also showed that traceability generates additional value when combined with other credence attributes.

Because traceability reduces information asymmetry and provides assurance regarding food origin, quality, and safety, consumers are expected to derive greater utility from a fast-food alternative that incorporates traceability information.

**H5.**  *The presence of food traceability positively influences consumers’ utility for a fast-food option.*

### 2.5. Price Premium and Fast-Food Choice

Price remains a central constraint in food choice because consumers evaluate product benefits against the financial sacrifice required to obtain them. This consideration is particularly important in the fast-food context, where affordability has traditionally been an important component of the value proposition.

Recent DCE evidence confirms that consumers do not evaluate health and sustainability independently of price. Fantechi et al. [12] found that a substantial consumer segment relied predominantly on price when choosing among foods varying in healthiness, sustainability, and naturalness. Likewise, evidence on sustainable-food labeling indicates that consumers may be willing to pay premiums for environmental attributes, but WTP is heterogeneous and remains constrained by the monetary trade-off involved [6].

In the present experiment, the monetary attribute did not represent the total menu price. Rather, respondents were shown a conventional fast-food item priced at TWD 80 and were asked to consider how much additional amount they would pay for alternatives containing selected attributes. Thus, the monetary variable is most appropriately described as a price premium: the experimental alternatives carried additional premiums of TWD 10, 20, or 30, whereas the conventional option required no additional payment (TWD 0). Consistent with consumer theory, increasing the required price premium is expected to reduce the utility associated with an alternative.

**H6.**  *A higher price premium negatively influences consumers’ utility for a fast-food option.*

### 2.6. Relative Valuation of Health, Sustainability, and Food-Assurance Attributes

Although the preceding hypotheses specify the expected direction of individual attribute effects, the main contribution of the present study is not merely to determine whether each attribute is valued positively, but to examine how strongly health-, sustainability-, and food-assurance attributes are valued relative to one another under a common monetary trade-off.

Recent studies increasingly demonstrate that health and sustainability are not necessarily substitutes or equally weighted objectives. Some consumers simultaneously value both dimensions, whereas others prioritize health, environmental impact, or price [12]. Likewise, combined nutrition and environmental labeling may generate interactions in consumer perceptions, highlighting the complexity of evaluating these dimensions jointly [13]. Given that the present study was designed to estimate the relative valuation of multiple food attributes rather than to test an a priori ordering among health-related, sustainability-related, and food-assurance attributes, no directional hypothesis was specified regarding which dimension would command greater willingness to pay. Instead, the following research question was proposed:

**RQ1.**  *How does consumers’ marginal willingness to pay vary across health-related, sustainability-related, and food-assurance attributes in fast-food choice?*

## 3. Materials and Methods

### 3.1. Study Design and Participants

This study employed a discrete choice experiment (DCE) to examine consumers’ preferences and willingness to pay for health-related, sustainability-related, and food-assurance attributes of fast-food options. The questionnaire retained the attribute structure and price-premium range established during prior instrument development and pretesting. A pretest was conducted from 1 to 15 May 2025 among consumers who had previously purchased fast food. A total of 83 pretest questionnaires were collected, of which 80 were valid, corresponding to a valid response rate of 96.4%. The pretest was used to examine respondents’ willingness to pay an additional amount for the selected fast-food attributes and to support the development of the price-premium levels used in the formal discrete choice experiment. No formal a priori power calculation was conducted. The initial operational recruitment target was approximately 400 respondents, reflecting the 22 questionnaire versions and an operational target of approximately 18 respondents per version. Additional questionnaires were collected to allow for incomplete or invalid responses. As a retrospective adequacy check, a commonly used rule-of-thumb benchmark for main-effects DCE designs [20], N > 500c/(t × a) yields an approximate minimum of 223 respondents for the present design, where c = 4 is the largest number of attribute levels, t = 3 is the number of choice tasks per respondent, and a = 3 is the number of alternatives per task. The final analytic sample of 401 respondents exceeded this heuristic minimum.

Participants were recruited using an online convenience-sampling approach, with the survey link distributed through social media channels. The main survey was administered online from 15 to 29 August 2025. Eligible respondents were consumers aged 21–60 years who had previously purchased fast food. A total of 458 questionnaires were collected. Forty-nine questionnaires were excluded during the initial screening because they contained incomplete responses, leaving 409 questionnaires for further data checking. Before model estimation, a structural integrity check of the long-format choice data was applied using an objective rule: each choice task had to contain three alternatives, exactly one selected alternative, and exactly one conventional alternative. Eight respondent records failed this structural-consistency rule and were excluded in full. These exclusions were based on the integrity of the choice-set structure rather than on respondents’ attribute preferences or model outcomes. The final analytic sample therefore comprised 401 respondents, corresponding to 87.6% of the questionnaires collected. The questionnaire also collected respondents’ sex, age, educational attainment, marital status, and monthly disposable expenditure on food and dining.

Before completing the choice tasks, respondents were provided with explanations of the five non-price attributes. The formal questionnaire also illustrated a conventional hamburger priced at TWD 80 and explained that fast-food options containing additional attributes could require an additional payment. Accordingly, the monetary attribute in the DCE represented an additional price premium rather than the total menu price.

### 3.2. Attributes and Attribute Levels

The five non-price attributes were retained from the prior questionnaire framework because they represented the main food-related dimensions examined in the study: environmental sustainability, food assurance, nutrition-related information, a general health-oriented cue, and an alternative-food option. The monetary attribute was specified as an additional price premium relative to the conventional fast-food option. The price-premium levels were subsequently developed with reference to the pretest on respondents’ willingness to pay an additional amount for these attributes. Before the pretest and main survey, the questionnaire items and attribute descriptions were reviewed through an expert-evaluation process. The experts were asked to assess whether the wording and descriptions were understandable and appropriate for the intended respondents. Based on this expert review, several wording and presentation adjustments were made to improve clarity and readability before the subsequent pretest and formal survey. The DCE contained five non-price attributes—carbon labeling, food traceability, nutrition labeling, a health-oriented cue, and an alternative-food option—and one monetary attribute representing the additional price premium (Table 1). The original attribute definitions were retained to preserve consistency with the survey administered to respondents. Because the health-oriented cue reflected the wording used in the original questionnaire rather than a standardized health claim, it was retained as originally operationalized and is interpreted conservatively throughout this study as a general health-related product cue. Rather, the original questionnaire presented a general ‘health orientation’ descriptor accompanied by a visual cue, rather than a specific nutritional or health claim (e.g., ‘low salt,’ ‘low saturated fat,’ or ‘low calorie’ Accordingly, the attribute is interpreted in this study only as a general health-oriented product cue. Its estimated coefficient and MWTP therefore represent the value associated with the presence of this general cue and should not be interpreted as willingness to pay for a specific nutritional property or as the effect of respondents’ individual health consciousness. For clarity, the experimental attributes represented the presence or absence of the corresponding information cue or label. The experimental alternatives varied in the presence or absence of nutrition and carbon-labeling cues rather than in specified numerical nutrient or carbon-emission values. Before the choice tasks, the questionnaire explained that nutrition labeling could contain information on energy, protein, fat, saturated fat, trans fat, sugar, carbohydrate, and sodium, while the carbon-label description referred generally to information concerning a product’s contribution to global warming and its carbon footprint. The estimated utilities and MWTP values should therefore be interpreted as valuations of the presence of these information cues, rather than as valuations of an objectively healthier or more environmentally friendly product.

**Table 1:** Attributes and levels used in the discrete choice experiment.

Attribute	Operational Description	Levels	Conceptual Dimension
Carbon label (CL)	Information concerning the product’s contribution to global warming and carbon footprint	Present/Absent	Sustainability-related
Traceability (TAP)	Information providing assurance regarding food origin, safety, and quality	Present/Absent	Food assurance
Nutrition label (NL)	Information on energy, protein, fat, saturated fat, trans fat, sugar, carbohydrate, and sodium	Present/Absent	Health-related
Health-oriented cue (HO)	General health-oriented product cue based on the original questionnaire’s “health orientation” descriptor; no specific nutrient or standardized health claim was presented.	Present/Absent	Health-related
Alternative-food option (SF)	Alternative foods involving innovations in food sources or production, including meat substitutes and vegetarian burgers	Present/Absent	Sustainability-related
Price premium (FUND)	Additional amount paid for the fast-food option	Experimental alternatives: TWD 10/20/30; conventional option: TWD 0	Economic

### 3.3. Discrete Choice Experimental Design

With five binary attributes and one four-level price attribute, the full factorial design comprised 128 possible profiles (2^5^ × 4 = 128). To reduce the number of profiles to a manageable set, an orthogonal array was generated using IBM SPSS Statistics (version 29; IBM Corp., Armonk, NY, USA), yielding 16 candidate profiles [21]. Of these, 12 profiles with a positive price premium (TWD 10, 20, or 30) were retained as experimental alternatives, while the zero-premium baseline was represented by the conventional status quo option.

The 12 experimental profiles were paired in all possible combinations, yielding 66 profile pairs (C(12,2) = 66). Each pair formed a choice task together with the conventional fast-food option. The 66 resulting choice tasks were then partitioned into 22 questionnaire versions, with three tasks assigned to each version. This partitioning limited the number of choice tasks completed by each respondent to three while allowing the broader set of profile combinations to be distributed across questionnaire versions. Accordingly, each respondent completed three choice tasks, and each task required a choice among two experimentally constructed fast-food alternatives and one conventional fast-food alternative. The profile pairings and task compositions varied across questionnaire versions. The questionnaire versions were allocated with the operational aim of achieving an approximately balanced number of respondents across versions rather than through a formally documented randomization procedure. In the final analytic sample, the number of respondents per questionnaire version ranged from 16 to 21, with an average of approximately 18.2 respondents per version. This distribution was therefore broadly consistent with the planned target of approximately 18 respondents per version. The initial candidate profiles were generated from an orthogonal array intended to reduce correlations among attribute levels. After restricting the design to the 12 profiles with positive price premiums, all possible pairwise combinations of these retained profiles were used, so that each retained experimental profile was paired once with every other retained profile across the full set of 66 choice tasks. No formal D-efficiency or A-efficiency criterion was calculated for the final paired and blocked design after profile restriction. Accordingly, the final design should be interpreted as an orthogonally derived, pragmatically reduced design with complete pairwise coverage of the retained experimental profiles, rather than as a formally D-efficient design.

### 3.4. Choice Task

Each choice task presented respondents with two experimentally constructed fast-food alternatives and one conventional fast-food alternative. The experimental alternatives varied in the presence or absence of the five non-price attributes and in the additional price premium. Respondents were instructed to select the option they preferred most. Thus, a “present” level indicated that the corresponding label or cue was displayed for that alternative, whereas an “absent” level indicated that it was not displayed. The choice tasks did not provide numerical nutrient profiles or quantitative carbon-emission values.

For example, the formal questionnaire presented Fast-food Options A and B constructed from combinations of carbon labeling, traceability, nutrition labeling, the health-oriented cue, the alternative-food option, and additional price premiums, while the conventional option required no additional payment. A reconstructed English-language representation of the attribute descriptions, visual cues, and an example choice task based on the retained questionnaire materials and the operationalization used in the present study is provided in the Supplementary Materials (Figures S1 and S2). An example of a choice task is presented in Table 2.

**Table 2:** Example of a choice task presented to respondents.

Attribute	Fast-Food Option A	Fast-Food Option B	Conventional Option
Carbon label	Absent	Absent	Absent
Traceability	Present	Present	Absent
Nutrition label	Absent	Absent	Absent
Health-oriented cue	Present	Absent	Absent
Alternative-food option	Absent	Present	Absent
Price premium	TWD 10	TWD 10	TWD 0
Choice	□	□	□

Note: □ indicates a checkbox for respondents to select their preferred alternative. Respondents were instructed to select one option per choice task.

### 3.5. Random Utility Framework

The analysis adopted a characteristics-based approach to consumer choice [22] and was grounded in random utility theory [23]. For respondent n, the utility derived from alternative j in choice task t was specified as

$$U_{njt}=V_{njt}+\varepsilon_{njt}$$

where $V_{njt}$ denotes the systematic component of utility and $\varepsilon_{njt}$ represents an unobserved stochastic component.

The systematic utility function was specified as

$$V_{njt}={\alpha ASC}_{njt}+\beta_{1}{CL}_{njt}+\beta_{2}{TAP}_{njt}+\beta_{3}{NL}_{njt}+\beta_{4}{HO}_{njt}+\beta_{5}{SF}_{njt}+\beta_{6}{FUND}_{njt}$$

where ASC denotes the alternative-specific constant associated with the conventional fast-food option; CL, TAP, NL, HO, and SF denote the five non-price attributes; and FUND denotes the additional price premium. For estimation, all five binary non-price attributes were dummy-coded consistently across alternatives (1 = present, 0 = absent). The conventional alternative therefore had CL = TAP = NL = HO = SF = 0 and FUND = 0, with ASC = 1 for the conventional alternative and ASC = 0 for the two experimental alternatives.

### 3.6. Conditional Logit Model

A conditional logit (CL) model was first estimated to examine the average effects of the attributes on consumer choice [23]. Under the CL specification, the probability that respondent n selects alternative j from choice set $C_{nt\;}$ is expressed as

$$P_{njt}=\frac{exp\left(V_{njt}\right)}{\sum_{k\in C_{nt}}exp\left(V_{nkt}\right)}$$

The CL model served as the baseline specification for testing the directional effects proposed in H1–H6 and for estimating the marginal utility associated with each attribute.

### 3.7. Random Parameter Logit Model

Because the conditional logit model assumes independence of irrelevant alternatives (IIA) and a homogeneous preference structure, a random parameter logit (RPL) model was additionally estimated to account for unobserved preference heterogeneity [24,25]. For respondent *n*, the random coefficient vector was specified as

$$\beta_{n}=\bar{\beta }+\eta_{n}$$

where $\bar{\beta }$ denotes the population mean coefficient vector and $\eta_{n}$ represents individual-specific deviations from the mean. The random parameters were assumed to follow normal distributions. In the RPL specification, the alternative-specific constant and the five non-price attributes were treated as mutually independent normally distributed random parameters, whereas the price-premium coefficient was specified as fixed. The model was estimated by simulated maximum likelihood using 500 deterministic Halton draws after discarding the first 20 sequence points [24]. The three repeated choices made by each respondent were treated as panel observations [24,25]. The estimated random-parameter standard deviations and corresponding *p*-values from the fitted RPL model are reported in Section 4.4. Because inference for variance components may be sensitive to boundary conditions, the statistical significance of these standard-deviation estimates should be interpreted cautiously.

### 3.8. Marginal Willingness to Pay

Marginal willingness to pay (MWTP) for attribute k was calculated as the negative ratio of its estimated coefficient to the coefficient of the monetary attribute [25]:

$$MWTP_{k}=\frac{-\beta_{k}}{\beta_{FUND}}$$

This measure represents the estimated additional amount respondents were willing to pay for the presence of a given fast-food attribute, holding other attributes constant. Approximate 95% confidence intervals for MWTP were calculated using the delta method based on the variance–covariance matrix of the conditional logit estimates.

### 3.9. Data Structure and Statistical Analysis

The 401 respondents each completed three choice tasks, yielding 1203 choice observations. Because each choice task contained three alternatives, the long-format estimation dataset comprised 3609 alternative-level records. For each retained choice task, exactly one alternative was coded as selected (1), whereas the two non-selected alternatives were coded as 0. Before estimation, the non-price attributes of the conventional alternative were harmonized with the same 0/1 dummy-coding scheme used for the experimental alternatives. The CL model was estimated by maximum likelihood, whereas the RPL model was estimated by simulated maximum likelihood in Python 3.13, using SciPy 1.17.0 for optimization and JAX 0.9.0.1 for automatic differentiation.

### 3.10. Ethical Considerations

The study was conducted in accordance with the Declaration of Helsinki and was approved by the Institutional Review Board of Chung Shan Medical University, Taiwan (IRB No. CS2-18159; approval date: 15 March 2025). Informed consent was obtained from all participants prior to participation.

## 4. Results

### 4.1. Respondent Characteristics

A total of 401 respondents were included in the final analysis. Females accounted for 53.9% of the sample and males for 46.1%. The largest age group was 21–30 years (30.9%), followed by those aged 31–40 years (23.4%), 41–50 years (22.9%), and 51–60 years (22.7%). Most respondents had completed college or university education (66.8%). Regarding marital and parental status, 51.6% were unmarried without children, followed by 36.9% who were married with children. For monthly disposable expenditure on food and dining, 32.4% reported TWD 6000 or less, followed by 29.2% reporting TWD 6001–8000 and 20.2% reporting TWD 8001–10,000. Overall, the sample included respondents across all four age groups, with a relatively high proportion of college-educated participants.

### 4.2. Conditional Logit Estimates and Hypothesis Testing

The conditional logit model indicated that all five non-price attributes significantly increased the utility associated with a fast-food option, whereas the price-premium coefficient was negative and statistically significant. Table 3 presents the coefficient estimates, standard errors, z-values, and corresponding hypothesis-testing results.

Nutrition labeling showed the largest positive coefficient (β = 0.645, *p* < 0.001), followed by the health-oriented cue (β = 0.601, *p* < 0.001) and traceability (β = 0.541, *p* < 0.001). Carbon labeling (β = 0.411, *p* < 0.001) and the alternative-food option (β = 0.400, *p* < 0.001) were also positively associated with choice utility. In contrast, the price premium had a significant negative effect (β = −0.051, *p* < 0.001), indicating that higher additional costs reduced the likelihood of selecting an option.

Accordingly, H1–H5 were supported, as nutrition labeling, the health-oriented cue, carbon labeling, the alternative-food option, and traceability were all positively associated with utility. H6 was also supported, as higher price premiums significantly reduced utility.

The alternative-specific constant was negative and statistically significant (β = −1.351, *p* < 0.001), indicating a residual tendency away from the conventional fast-food alternative after accounting for the observed attributes.

### 4.3. Marginal Willingness to Pay and Relative Attribute Valuation

Marginal willingness to pay was calculated using the ratio of each attribute coefficient to the negative price-premium coefficient. All five non-price attributes yielded positive MWTP estimates, indicating positive estimated willingness to pay for the presence of each attribute.

The highest MWTP point estimate was observed for nutrition labeling (TWD 12.63), followed by the health-oriented cue (TWD 11.76) and traceability (TWD 10.59). Lower, but still positive, point estimates were observed for carbon labeling (TWD 8.05) and the alternative-food option (TWD 7.84). In response to RQ1, the MWTP point estimates were highest for the two health-related attributes, followed by the food-assurance attribute, whereas the two sustainability-related attributes had lower—but still positive—point estimates. These results are summarized in Table 4. The corresponding delta-method 95% confidence intervals excluded zero for all five attributes.

### 4.4. Random Parameter Logit Results and Preference Heterogeneity

The random parameter logit model produced coefficient patterns broadly consistent with those obtained from the conditional logit model. Carbon labeling, traceability, nutrition labeling, the health-oriented cue, and the alternative-food option all retained positive and statistically significant mean coefficients, whereas the price-premium coefficient remained negative and significant. The RPL estimates and random-parameter standard deviations are presented in Table 5.

The RPL model achieved a log-likelihood of −1008.786 and an AIC of 2043.600, compared with a log-likelihood of −1025.329 and an AIC of 2064.700 for the conditional logit model, suggesting improved model fit after allowing for random preference variation.

None of the random-parameter standard deviations associated with carbon labeling, traceability, nutrition labeling, the health-oriented cue, or the alternative-food option was statistically significant. The standard deviation of the ASC, however, was significant (σ = 1.683, *p* < 0.001), indicating heterogeneity in respondents’ preference for the conventional fast-food alternative after accounting for the observed attributes.

**Table 5:** Random parameter logit estimates and preference heterogeneity.

Variable	Mean Coefficient	SE	z-Value	SD of Random Parameter	*p*-Value for SD
ASC	−2.257	0.333	−6.770 ***	1.683	<0.001
Carbon label (CL)	0.427	0.121	3.520 ***	0.175	0.780
Traceability (TAP)	0.590	0.128	4.630 ***	0.235	0.669
Nutrition label (NL)	0.686	0.126	5.440 ***	0.216	0.790
Health-oriented cue (HO)	0.626	0.107	5.870 ***	0.194	0.771
Alternative-food option (SF)	0.428	0.103	4.170 ***	0.369	0.263
Price premium (FUND)	−0.053	0.007	−7.590 ***	—	—

Model statistics: Log likelihood = −1008.786; McFadden pseudo-R^2^ = 0.237; AIC = 2043.600; 500 Halton draws; 401 panel groups; 1203 choice observations. Note: Price premium was specified as a fixed parameter. *** *p* < 0.001.

## 5. Discussion

### 5.1. Principal Findings

This study examined how consumers trade off health-related, sustainability-related, food-assurance, and monetary attributes when choosing among fast-food options. The conditional logit results showed that all five non-price attributes contributed positively to choice utility, whereas a higher price premium reduced utility. At the point-estimate level, nutrition labeling and the health-oriented cue yielded the highest MWTP estimates, followed by traceability, whereas carbon labeling and the alternative-food option showed lower—but still positive—estimates.

The MWTP estimates provide a more direct indication of the relative economic value attached to these attributes. Nutrition labeling yielded the highest point estimate (TWD 12.63), followed by the health-oriented cue (TWD 11.76), traceability (TWD 10.59), carbon labeling (TWD 8.05), and the alternative-food option (TWD 7.84). At the point-estimate level, health-related attributes showed higher monetary valuations than sustainability-related attributes within the fast-food choice context. However, this ordering should be interpreted descriptively because the present analysis did not formally test pairwise statistical differences among the MWTP estimates.

### 5.2. Balancing Health, Food Assurance, and Sustainability

The relatively high valuation of nutrition labeling is consistent with recent evidence showing that nutrition information can improve consumers’ understanding of nutritional quality and support healthier food choices [2,3]. Kelly et al. [3], in a large systematic review of front-of-pack nutrition labeling, concluded that nutrition labels can improve consumers’ understanding of nutritional quality and the healthfulness of food choices and purchases, particularly when interpretive formats are used. Similarly, Abbas and Hatch [2] found that restaurant nutrition information can influence consumer decisions, although its effectiveness varies across consumer groups and contexts. The present findings extend this evidence to a multi-attribute fast-food choice setting, suggesting that nutrition information remained highly valued even when respondents simultaneously considered environmental, food-assurance, and price attributes.

The relatively high MWTP associated with the health-oriented cue provides additional evidence that health-related positioning can generate consumer value. Nevertheless, this result requires careful interpretation because the health-oriented cue in the original DCE represented a binary product-level signal rather than a validated individual-level measure of health consciousness. The finding therefore indicates that respondents valued the presence of a health-related signal in the choice task; it should not be interpreted as evidence that respondents with greater personal health orientation necessarily exhibited stronger preferences.

Traceability also showed a comparatively high valuation relative to the sustainability-related attributes, reinforcing the conceptual distinction between food assurance and direct sustainability claims. Traceability primarily reduces information asymmetry concerning origin, safety, quality, and supply-chain transparency rather than directly communicating health or environmental performance. The positive valuation of traceability is consistent with the broader literature. Tran et al. [7], in a meta-analysis of 72 studies, reported a substantial and increasing consumer WTP for food traceability, with an average premium of approximately 32% across the studies examined.

Taken together, these findings suggest that consumers may not evaluate health, sustainability, and food assurance as interchangeable benefits. Instead, they appear to assign distinct values to different forms of information. In the present study, nutrition-related and health-related attributes showed higher MWTP point estimates, while traceability also showed a positive and comparatively high valuation as a food-assurance attribute.

### 5.3. Sustainability Attributes and Preference Patterns

Although the two sustainability-related attributes showed lower MWTP point estimates than the health-related attributes, both carbon labeling and the alternative-food option significantly increased choice utility. Sustainability should therefore not be interpreted as unimportant in fast-food decisions. Rather, the results indicate that the sustainability-related attributes generated positive consumer value, but their monetary valuations were comparatively lower within the set of attributes examined.

The positive effect of carbon labeling is consistent with recent experimental evidence from fast-food settings. Wolfson et al. [4] found that climate-impact menu labels altered fast-food ordering choices among U.S. adults, with high-climate-impact labels producing particularly pronounced shifts away from higher-impact items. More recently, Wolfson et al. [5] showed that traffic-light-style ecolabels reduced the greenhouse-gas emissions associated with full meal orders in both burger- and sandwich-focused fast-food contexts and, in one restaurant setting, also improved nutritional quality. These studies demonstrate that environmental information can influence restaurant food choices even when sustainability is not the traditional basis of fast-food positioning.

The alternative-food option received the lowest MWTP point estimate among the five non-price attributes, although its coefficient remained positive and statistically significant. In the RPL model, however, its random-parameter standard deviation was not statistically significant (σ = 0.369, *p* = 0.263). Thus, the present analysis did not detect significant unobserved heterogeneity in preferences for the alternative-food option under the assumed normal random-parameter specification.

This result differs somewhat from prior studies that have reported substantial heterogeneity in consumer acceptance of meat alternatives. Chen et al. [8] found that nutritional and environmental information significantly influenced WTP for plant-based and cultured meat, while Jiang et al. [9] reported that environmental information increased preferences and WTP for selected prepared plant-based meat products and identified variation related to food-technology neophobia. The contrast suggests that preference heterogeneity may depend on product context, observed consumer characteristics, or the statistical representation used to model latent segments. Market availability may also help contextualize the comparatively lower valuation of the alternative-food option observed in the present study. Plant-based fast-food offerings may differ in availability across markets. McDonald’s Taiwan introduced the McPlant as a limited-time plant-based burger in 2022 [26], whereas McDonald’s UK currently offers a vegan-certified McPlant [27]. These cross-market differences provide contextual background only and should not be interpreted as explaining the valuation observed in the present study, because familiarity, market exposure, prior consumption experience, and perceived availability were not measured. Future research could examine these factors as potential moderators of preference.

The absence of statistically significant random-parameter standard deviations for the five non-price attributes should not be interpreted as proof that all respondents held identical preferences. Factors such as familiarity, perceived naturalness, taste expectations, technology acceptance, or food neophobia were not directly modeled as sources of heterogeneity and should be examined in future research using observed interactions or latent-class approaches.

### 5.4. Theoretical and Practical Implications

The study provides several contributions to consumer food-choice research and the evaluation of food-product attributes. First, it evaluates health-related, sustainability-related, food-assurance, and monetary attributes simultaneously within a common discrete choice framework. Much previous research has examined nutrition labels, environmental labels, traceability, or alternative foods separately. By placing these attributes within the same choice task, the present study provides evidence on their relative valuation under a common monetary trade-off.

Second, the results indicate that sustainable healthy food choice should not necessarily be conceptualized as a simple opposition between personal health and environmental sustainability. Instead, consumers may assign value to both dimensions while weighting them differently. Recent DCE evidence similarly shows substantial heterogeneity in how consumers balance healthiness, sustainability, naturalness, and price. Fantechi et al. [12], for example, identified consumers who valued health and sustainability jointly, consumers who prioritized health, and a substantial segment whose decisions were driven primarily by price. The present results complement this evidence by showing that, at the aggregate level, health-related attributes received the highest MWTP point estimates while sustainability-related attributes continued to provide positive utility.

Third, the results reinforce the value of distinguishing food assurance from sustainability. Traceability generated a relatively high MWTP despite not being classified as either a health or environmental attribute. This finding suggests that consumers may value transparency, origin, safety, and quality assurance as a separate dimension of food choice rather than simply as an extension of sustainability.

From a practical perspective, the results suggest that fast-food operators seeking to enhance consumer value may benefit from making nutrition information and health-related cues salient and easy to interpret. Traceability information may provide an additional source of value by strengthening perceptions of origin, safety, and transparency. Sustainability information should not be omitted merely because its MWTP estimates were comparatively lower: both carbon labeling and alternative-food options positively influenced choice utility. Recent experiments further suggest that label design and interpretability can influence responses to environmental information, underscoring the importance of testing simple and salient formats in fast-food settings [4,5,13].

For alternative-food options, the present RPL specification did not identify significant attribute-level heterogeneity, but prior evidence indicates that information content, product framing, and taste-related judgments can shape responses to meat alternatives and other fast-food alternatives [8,9,18,19]. Communication that combines environmental information with clearly understandable health, product, and quality information may therefore help consumers evaluate unfamiliar alternatives. Nevertheless, the present study did not experimentally compare communication strategies, and these recommendations should therefore be regarded as implications for future testing rather than established causal effects.

### 5.5. Limitations and Future Research

Several limitations should be acknowledged. First, participants were recruited through social media using an online convenience-sampling approach. Although the eligibility criteria ensured that included respondents had prior fast-food purchasing experience, the non-probability sampling method may have introduced self-selection bias, and the sample should not be interpreted as nationally representative of all Taiwanese consumers. Future studies could use probability-based or quota-based sampling to strengthen population-level generalizability.

Second, the study relied on stated choices in a hypothetical discrete choice experiment rather than observed purchasing behavior. Although DCEs are well suited to estimating trade-offs and MWTP, hypothetical choices may not fully reproduce decisions made in actual restaurant settings, where taste, time pressure, brand familiarity, social context, and budget constraints may also influence behavior. Field experiments or studies using actual purchase data could provide useful external validation.

Third, the study examined a defined set of five non-price attributes and one monetary attribute. Other factors relevant to fast-food choice—including taste, convenience, brand, portion size, familiarity, sensory expectations, and prior experience—were not included in the experimental design. Their omission means that the estimated attribute valuations should be interpreted relative to the choice set used in the present study rather than as comprehensive measures of all determinants of fast-food choice.

Fourth, each respondent completed three choice tasks drawn from 22 questionnaire versions based on the experimental profile design. This approach reduced respondent burden while distributing the broader set of profile combinations across questionnaire versions. Nevertheless, future studies could employ formally optimized efficient designs and additional choice tasks per respondent to examine the robustness and precision of preference estimates.

Fifth, the health-oriented cue requires particular interpretive caution. Although the questionnaire described health orientation in terms of personal attitudes, beliefs, and behaviors, the DCE operationalized it as a binary product-level attribute. The present manuscript therefore treats it as a health-oriented cue rather than a validated psychometric construct. Because the cue was not operationalized as a specific standardized product claim, its coefficient should be interpreted as the utility associated with the presence of the original general health-oriented descriptor. Future DCEs should separate individual health consciousness from experimentally manipulated, clearly specified health claims or product attributes.

Sixth, the RPL model did not identify statistically significant random-parameter heterogeneity for any of the five non-price attributes. This finding should therefore be interpreted as an absence of detectable heterogeneity under the present model specification rather than as evidence of homogeneous preferences across all respondents. Future research could examine alternative distributional assumptions, observed interactions, latent-class models, or other mixture approaches to determine whether distinct consumer segments differ in their trade-offs among health, sustainability, food assurance, and price.

Finally, the study was conducted among respondents in Taiwan, and food culture, familiarity with labeling systems, environmental awareness, and acceptance of alternative foods may differ across countries.

## 6. Conclusions

This study used a discrete choice experiment to examine how Taiwanese respondents trade off health-related, sustainability-related, food-assurance, and monetary attributes when choosing among fast-food options. Nutrition labeling, a health-oriented cue, carbon labeling, food traceability, and an alternative-food option all contributed positively to choice utility, whereas a higher price premium reduced utility. These findings indicate that respondents assigned positive value to health-, sustainability-, and food-assurance attributes while remaining sensitive to the additional monetary cost associated with these benefits.

The MWTP point estimates revealed a descriptive ordering in the valuation of the examined attributes. Nutrition labeling yielded the highest point estimate (TWD 12.63), followed by the health-oriented cue (TWD 11.76), traceability (TWD 10.59), carbon labeling (TWD 8.05), and the alternative-food option (TWD 7.84). At the point-estimate level, the two health-related attributes showed higher monetary valuations than the two sustainability-related attributes, while food traceability also showed a positive and comparatively high valuation as a distinct food-assurance attribute. Because pairwise statistical differences among the MWTP estimates were not formally tested, this ordering should be interpreted descriptively. At the same time, the positive utility associated with carbon labeling and the alternative-food option indicates that sustainability remained relevant to fast-food choice.

The random parameter logit results further showed that average preference patterns were broadly consistent with the conditional logit estimates. No statistically significant random-parameter heterogeneity was detected for the five non-price attributes, whereas significant heterogeneity was observed for the conventional alternative-specific constant. Taken together, the findings support examining health, sustainability, food assurance, and price jointly within a common food-choice framework rather than treating them as separate influences. Within the present study sample, nutrition labeling, health-oriented cues, traceability, carbon labeling, and alternative-food options were each associated with positive utility, although their estimated valuations differed descriptively. Future research using cross-cultural, field-based, efficiently designed, or latent-class discrete choice studies could further examine how these trade-offs vary across consumer segments and market settings.

## Figures and Tables

**Table 3:** Conditional logit model estimates and hypothesis-testing results.

Variable	Coefficient (β)	SE	z-Value	Hypothesis
Carbon label (CL)	0.411	0.105	3.920 ***	H3 supported
Traceability (TAP)	0.541	0.106	5.102 ***	H5 supported
Nutrition label (NL)	0.645	0.102	6.312 ***	H1 supported
Health-oriented cue (HO)	0.601	0.093	6.442 ***	H2 supported
Alternative-food option (SF)	0.400	0.092	4.362 ***	H4 supported
Price premium (FUND)	−0.051	0.005	−9.472 ***	H6 supported
ASC	−1.351	0.204	−6.622 ***	—

Note: ASC = alternative-specific constant for the conventional fast-food option; SE = standard error. *** *p* < 0.001.

**Table 4:** Marginal willingness to pay for fast-food attributes.

Attribute	MWTP Point Estimate (TWD)	95% CI (TWD)	Conceptual Dimension
Nutrition label	12.63	8.04–17.21	Health-related
Health-oriented cue	11.76	7.71–15.81	Health-related
Traceability	10.59	6.10–15.08	Food assurance
Carbon label	8.05	3.79–12.32	Sustainability-related
Alternative-food option	7.84	4.15–11.52	Sustainability-related

Note: MWTP was calculated as the negative ratio of the attribute coefficient to the price-premium coefficient. Approximate 95% confidence intervals were calculated using the delta method based on the conditional-logit variance–covariance matrix. No pairwise statistical tests were conducted among the MWTP point estimates.

## Data Availability

The complete dataset is not publicly available due to ethical and privacy considerations related to the protection of research participants. The data presented in this study are available from the corresponding author upon reasonable request.

## Supplementary Materials

The following supporting information can be downloaded at https://www.mdpi.com/article/10.3390/foods15203679/s1: Figure S1: Reconstructed English-language representation of the five non-price attributes used in the discrete choice experiment; Figure S2: Reconstructed example of a discrete choice task based on the experimental design.
