# Supplementary material for: Balancing Health, Sustainability, and Food Assurance in Fast-Food Choice: A Discrete Choice Experiment in Taiwan
Source: Foods. 2026 Oct 16;15(20):3679. doi: 10.3390/foods15203679 (PMC13704544; doi:10.3390/foods15203679)
Supplement: Supplementary file 1 [file foods-15-03679-s001.zip › foods-4623909-supplementary.pdf]

Supplementary Materials

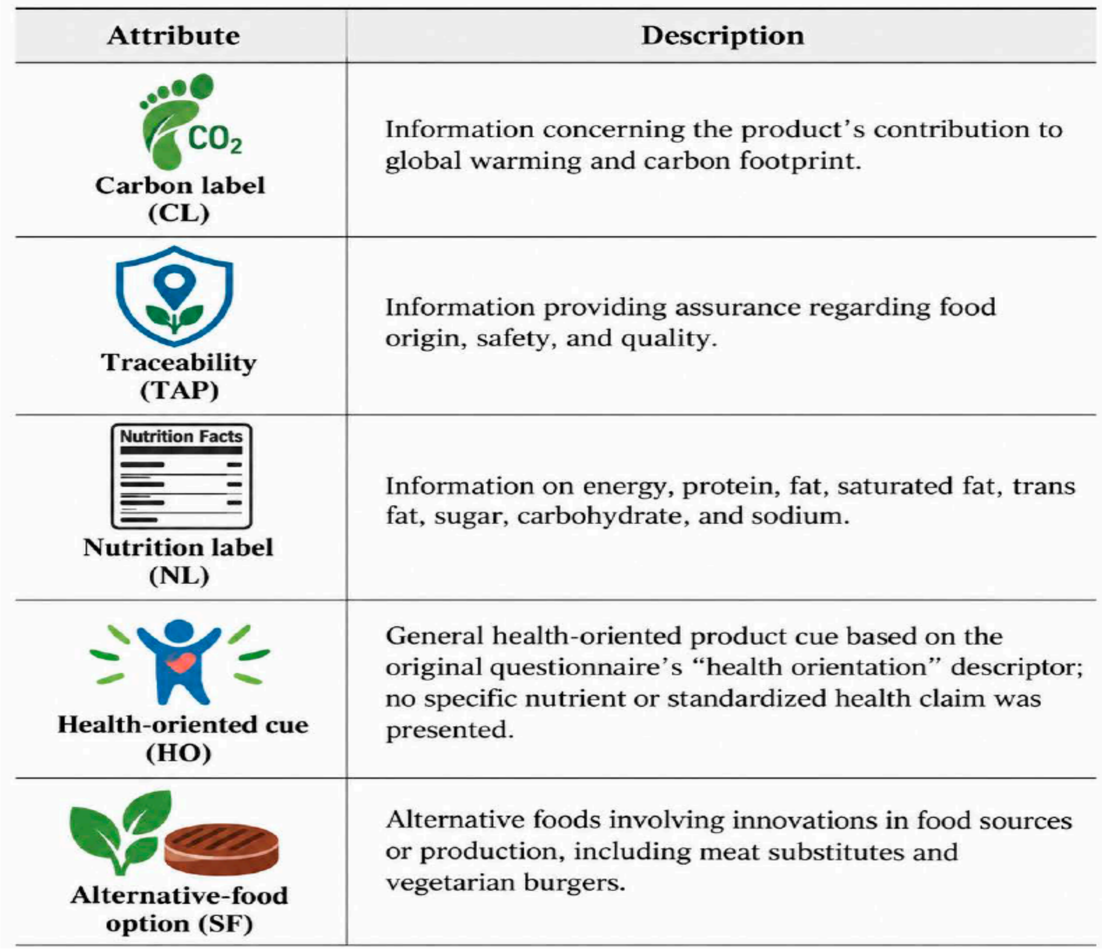

Attribute	Description
Carbon label (CL)	Information concerning the product's contribution to global warming and carbon footprint.
Traceability (TAP)	Information providing assurance regarding food origin, safety, and quality.
Nutrition label (NL)	Information on energy, protein, fat, saturated fat, trans fat, sugar, carbohydrate, and sodium.
Health-oriented cue (HO)	General health-oriented product cue based on the original questionnaire's "health orientation" descriptor; no specific nutrient or standardized health claim was presented.
Alternative-food option (SF)	Alternative foods involving innovations in food sources or production, including meat substitutes and vegetarian burgers.

Figure S1. Reconstructed English-language representation of the five non-price attributes used in the discrete choice experiment. **Note:** This figure was reconstructed from the retained questionnaire materials and aligned with the operational definitions used in the present manuscript.

Please select one option (choose 1).

Attribute	Fast-food Option A	Fast-food Option B	Conventional Option
Carbon label	Absent	Absent	Absent
Traceability	Present	Present	Absent
Nutrition label	Absent	Absent	Absent
Health-oriented cue	Present	Absent	Absent
Alternative-food option	Absent	Present	Absent
Price premium	TWD 10	TWD 10	TWD 0
Choice	☐	☐	☐

Figure S2. Reconstructed example of a discrete choice task based on the experimental design. **Note:** The choice-task structure was reconstructed from the retained questionnaire materials and analytic coding. Respondents selected one option from two experimentally constructed fast-food alternatives and one conventional option.
